# Chemical Tools of *Octopus maya* during Crab Predation Are Also Active on Conspecifics

**DOI:** 10.1371/journal.pone.0148922

**Published:** 2016-02-19

**Authors:** Dawrin Pech-Puch, Honorio Cruz-López, Cindy Canche-Ek, Gabriela Campos-Espinosa, Elpidio García, Maite Mascaro, Carlos Rosas, Daniel Chávez-Velasco, Sergio Rodríguez-Morales

**Affiliations:** 1 Unidad de Química-Sisal, Facultad de Química, Universidad Nacional Autónoma de México, Sisal, Yucatán, México; 2 Departamento de Bioquímica, Facultad de Química, Universidad Nacional Autónoma de México, México, D.F., México; 3 Unidad Multidisciplinaria Docencia e Investigación, Facultad de Ciencias, Universidad Nacional Autónoma de México, Sisal, Yucatán, México; 4 Centro de Graduados e Investigación Química, Instituto Tecnológico de Tijuana, Tijuana, Baja California Norte, México; The Evergreen State College, UNITED STATES

## Abstract

*Octopus maya* is a major socio-economic resource from the Yucatán Peninsula in Mexico. In this study we report for the first time the chemical composition of the saliva of *O*. *maya* and its effect on natural prey, i.e. the blue crab (*Callinectes sapidus*), the crown conch snail (*Melongena corona bispinosa*), as well as conspecifics. Salivary posterior glands were collected from octopus caught by local fishers and extracted with water; this extract paralyzed and predigested crabs when it was injected into the third pereiopod. The water extract was fractionated by membrane ultrafiltration with a molecular weight cut-off of 3kDa leading to a metabolic phase (>3kDa) and a neurotoxic fraction (<3kDa). The neurotoxic fraction injected in the crabs caused paralysis and postural changes. Crabs recovered to their initial condition within two hours, which suggests that the effects of the neurotoxic fraction were reversible. The neurotoxic fraction was also active on *O*. *maya* conspecifics, partly paralyzing and sedating them; this suggests that octopus saliva might be used among conspecifics for defense and for reduction of competition. Bioguided separation of the neurotoxic fraction by chromatography led to a paralysis fraction and a relaxing fraction. The paralyzing activity of the saliva was exerted by amino acids, while the relaxing activity was due to the presence of serotonin. Prey-handling studies revealed that *O*. *maya* punctures the eye or arthrodial membrane when predating blue crabs and uses the radula to bore through crown conch shells; these differing strategies may help *O*. *maya* to reduce the time needed to handle its prey.

## Introduction

The Mexican red octopus, *Octopus maya*, is a large endemic species of the Yucatan Peninsula where it is of social and economic importance [1]. It occurs mainly in shallow waters near the shore between 2 and 25 m depth along the continental shelf of the Yucatan Peninsula. *Octopus maya* is one of the most promising octopus species for aquaculture because holobenthic hatchlings become benthic juveniles only 7 to 10 days after hatching [2]. Considerable advances have occurred during the short history of *O*. *maya* aquaculture [3]; for instance, new outdoor tanks have been designed, as well as a successful diet that allows animals to reach 250 g of body weight within 120 days post hatching [2].

Recent studies have demonstrated that octopuses are voracious and active carnivores; they have a complex feeding behavior which depends on their victim, from the detection to the consumption of prey [4, 5]. Octopods feed on a variety of benthic prey that includes crustaceans, gastropods, bivalves, fishes, and birds, although many species prefer decapod crustaceans such as crabs, and some bivalves [5]. However, defensive structures of crabs such as sharp chelae represent a problem for the predator. Therefore, octopuses need a strategy to avoid body damage from cutting chelae during handling [6]. A cautious handling behavior has been described in some species of cephalopods [7] but it has not been studied in *O*. *maya* when feeding on various types of prey such as gastropods and crustaceans. The calcareous surface and chitinous exoskeleton of gastropods and crustaceans respectively protect them against predator attacks and are likely to represent difficulties for octopuses. Foraging studies have shown that octopods envelop their prey with their arms and web; the prey is strongly held by the circumoral suckers of the octopus and then carried to the mouth. At this point, the octopus injects saliva from the posterior salivary glands (PSGs) into the prey [4, 5, 8]. However, the mechanism by which *O*. *maya* subdues and kills its prey remains unclear. To date, studies of prey handling behavior in octopods have revealed some of the mechanisms by which octopuses introduce saliva into their prey. These mechanisms include hole-boring or drilling [9, 10] and eye puncture [11], although arthrodial membrane puncture has also been suggested [11, 12]. The site of injection of the saliva also appears to be influenced by both prey and octopus size [8, 4]. However, the mechanism used by *O*. *maya* to handle its prey remains unexplained. A study of predatory events and handling strategies developed by *O*. *maya* may help in the design of artificial baits for the fishery and for aquaculture.

Saliva is a venomous fluid created in the octopus PSG and it can be used for the acquisition of food, for defense and to reduce competition [13]. The saliva of octopuses has two major functions: paralysis and pre-digestion of prey [14–16]. It consists of biogenic amines (serotonin, octopamine, and tryptamine), neuropeptides (tachykinins) [17] and some metabolic enzymes, e.g. trypsin, chymotrypsin, and chitinase [18]. Molecular studies have also revealed the presence of chitinase, peptidase S1, antigen 5, pathogenesis-related peptide (PR-1), phospholipase A2 (PLA2), and six novel types of peptides [19]. The pre-digestive function of the saliva is due to the metabolic enzymes that allow the detachment of exoskeletal tissue and thereby facilitate the extraction of muscle [11]. The paralyzing activity of saliva was first attributed to tyramine (4-hydroxyphenethylamine) [20] and recently to α- and β-cephalotoxins, two potent glycoproteins (91.2 and 33.9 kDa MW, respectively) [21]. Venoms of cephalopods, including octopuses, share similar biochemical compositions, suggesting a shared ancestry [19].

Cannibalism is common in octopods [22], e.g. more than 10% of *O*. *maorum* and *O*. *tehuelchus* diets result from cannibalism. Cannibalistic behavior in octopuses might be related to their high protein demand, voracious feeding, high density and the lack of social behavior [23]. Cannibalism is also important in octopods since cephalopods have limited energy storage. In case of food depletion, octopods can adjust to environmental conditions by reducing their numbers. Additionally, octopus can kill conspecifics by strangulation [24] or presumably by biting individuals, which may result in venom injection [25], although the latter killing technique has not been examined. *Octopus maya* has also shown cannibalism when reared in aquaria [2], but there are no detailed records on such behavior.

The aims of this study are: 1) to determine the chemical substances that *O*. *maya* uses in the predation of blue crabs and to apply those substances on conspecifics to demonstrate the biochemical mechanism of cannibalism; and 2) to describe the context in which saliva is normally used when octopuses feed on crabs of different sizes, and to identify the main site of injection. We separated saliva into metabolic and reversible neurotoxic fractions, both toxic to their prey and to conspecifics. The paralyzing effect of saliva on blue crabs was caused by amino acids, while a relaxing activity was induced by serotonin in both crabs and *O*. *maya* subadults. We have also demonstrated for the first time that saliva in octopods might be used for defense and for competition against other predators.

## Materials and Methods

### Ethics Statement

This study followed the Guide for the Care and Use of Cephalopods in Research [26, 27] and our protocols were approved by the Experimental Animal Ethics Committee of the Faculty of Chemistry at Universidad Nacional Autónoma de México (Permit Number: Oficio/FQ/CICUAL/099/15).

Animals were anesthetized in cold sea water (2°C) under hyper-oxygenation as recommended for tropical cephalopod species. All efforts were made to minimize stress of the animals; a minimum number of animals were killed by an incision between the eyes.

### Collection and Maintenance of Experimental Animals

Octopus were collected from Sisal harbor (20°51’20.26”N and 90°1’49.38”W, 21°13’59.42”N and 89°53’22.52”W) by traditional fishing methods called "jimba y gareteo" where fishermen attach on each side of the ship a 5 m bamboo stick tied to a rope with a weight of steel and with crab as bait. Live octopus are caught and pulled up on board, where juvenile octopus can be returned to the sea without damage (S1 video).

In total, 100 octopus were captured during four collection trips and placed in a 200-L tank with seawater, changing the seawater every hour until arrival at the dock. In the laboratory, animals were individually placed in 80-L tanks with constant aeration and a flow-through seawater system under the following conditions: water temperature 24.65–29.23°C, salinity 35.71–35.72‰, dissolved oxygen 4.93–5.23 mg/L, and pH 7.61–7.79. Body mass was used as a criterion to define two octopus size categories: ‘small’ (≤ 450 g) and ‘large’ (≥ 450 g), representing subadult and adult stages respectively [28]. Total weight (g) was measured with a balance scale. Octopus were collected five days before trials commenced, with three days for acclimation and two days for fasting.

Blue crabs (*Callinectes sapidus*) were selected as target prey owing to a greater selectivity of the octopus towards crustaceans [11, 14]. In total, 150 crabs were randomly collected from the soft bottom of the intertidal shallow shores of Sisal. The carapace width (CW) was measured to include the lateral spines and it was used to arbitrarily classify crabs by size [28]. Crabs with < 12 cm CW were classified as small, and crabs with > 12 cm CW as large. The crabs were placed in a 200-L tank with aeration and a flow-through seawater system. The surface of the carapace of the crabs was examined for any damage; crabs in good condition were then numbered and individually placed in 100-L tanks with aeration and a flow-through seawater system.

Thirty crown conch snails (*Melongena corona bispinosa*) were collected from the Sisal coast and placed in a 100-L tank with aeration and a flow-through seawater system. Each individual was assigned a number; each shell was examined with a stereoscopic microscope, and imperfections or damage were recorded. All snails were maintained in a 200-L tank with aeration and a flow-through seawater system.

### Bioguided Separation of *Octopus maya* Saliva

#### Reagents and Instrumentation

All reagents were ACS grade, and solvents were HPLC grade. Analysis and separation were performed on a High Performance Liquid Chromatography system (HPLC), equipped with a Polaris 211 dual pump solvent delivery system, 314 UV detector (Agilent Technologies, USA), and Evaporative Light Scattering Detector (ELSD, Polymer Ice Inc. USA) connected to a workstation with Galaxy 1.9.3 software. This system was used in two conditions:

1. Analytical: A Luna reverse-phase C18 column (250 × 4.6 mm, 5 μm, Phenomenex Inc. USA) was used. The mobile phase, 0.1% trifluoroacetic acid (A) and acetonitrile (B), was used in a gradient elution as follows: 0–8 min, 0% to15% B; 8–25 min, 15% to 35% B; 25–30 min, 35% to 100% B; and finally 30–35 min, 100% B to 100% A. The mobile-phase flow rate was 1 mL/min and the injection volume was 20 μL (1 mg/mL of each fraction analyzed). Elution was monitored at 215 nm in the UV detector, and ELSD conditions were as follows: nebulization temperature 40°C, evaporation temperature 80°C, and nitrogen flow 2.0 mL/min.
2. Semipreparative: A Luna reverse-phase C18 column (250 × 100 mm, 10 μm, Phenomenex Inc. USA) was used. Mobile phase and gradient were as above, flow rate was 3 mL/min and the injection volume was 20 μL (5 mg/mL of each fraction). Elution was monitored at 215 nm.

Amino acid profile was determined in a Waters HPLC system equipped with a 1525 dual pump, 2475 Fluorescence and 2475 UV detectors, using the conditions established in the AccQ-Tag Protocol from Waters [29].

^1^H and ^13^C NMR spectra were recorded on a Bruker Avance III 400 spectrometer (Bruker Inc. Mass. USA) operating at 400 MHz and at 125.758 MHz respectively, and using a nitrogen cryogenic probe. Standard Bruker Topspin 2.1 software was used. All experiments were performed at 22°C in deuterochloroform (Sigma, 151856) solution with the solvent peak as an internal standard set at 7.27 ppm (^1^H) or 77.0 (^13^C) vs TMS respectively. A first-order analysis was applied, and first-order multiplets or apparent first-order multiplets were denoted as follows: s, singlet; d, doublet; dd, double-doublet; t, triplet. J-values were extracted directly from the splitting in the spectrum and were not optimized. Spectral assignments were based not only on the usual chemical shift rules and coupling patterns but especially on routine 2D-correlations such as COSY (homonuclear H,H J-correlations), HSQC (single bond C,H ^1^J-correlations), and HMBC experiments (multiple-bond C,H ^3^J-correlations).

Electron impact mass spectra with direct insertion were produced on a mass spectrometer HP-5975C (Agilent Tech). The temperature of the transfer line to the MS was set to 300°C. Ionization of Compounds were ionized by electron impact at 70eV. The ion source temperature was 230°C and the quadrupole temperature was 150°C. Full scans were acquired from m/z 40 to m/z 500. Data were processed with MSD-Chemstation Software (MSD ChemStation E.02.00.493; Agilent Technologies).

#### Saliva extraction from Posterior Salivary Glands from fishery byproducts

Posterior salivary glands (PSGs; 500 pairs, ~503 g; S1 powerpoint) were collected from eviscerated octopus caught by local fishers between August and December 2014. The glands were deposited in 1-L plastic bottles (Nalgene) and stored in an ultrafreezer at -70°C (REVCO, thermo-Science, USA) until they were lyophilized, milled to fine dust (110 g), extracted with water (1:10, v/v), and centrifuged (3857 rcf / 20 min / 23°C). The supernatant was collected and lyophilized, and then a sample was reconstituted in water and tested to determine its effect on the blue crab.

#### Bioassay

All extracts and fractions were evaluated in an *in vivo* neurotoxic bioassay on the ghost crab *Ocypode quadrata*. Crabs were collected from the coastal dune near the port of Sisal and kept in a container with sand. The test fractions were prepared with 10 mg of extract or fraction diluted in distillated water to obtain solutions with concentrations of 0.1, 1, 10, 100 and 1000 μg/mL; 100 μL water was used as the negative control. A neurotoxic assay was performed by injecting 100 μL of the solution into the third pereiopod (ambulatory leg) of the ghost crab [30]. Any effect on the crab, e.g. shaking, trembling or death, was recorded.

Initially, different crustaceans were used, from marine crabs (*Libinia dubia*, *Menippe* spp.) and blue crabs (*C*. *sapidus*) to semi-terrestrial species such as the fiddler crab (*Uca* spp.) (S2 video). In all cases it was possible to determine the effect of the crude extract; however, the neurotoxic effects were more evident in the ghost crab.

#### Isolation of metabolic and neurotoxic phases

A diluted crude PSG extract (15 mL, 800 mg) was centrifuged through an ultrafiltration membrane (Amicon, Millipore, 1 h, 3857 rcf, 25°C) with a 3 kDa molecular weight cut off (MWCO), the metabolic phase (< 3 kDa) was concentrated to 250 μL, and the filtrate (> 3kDa) was lyophilized (400 mg) and evaluated in the bioassay. Separation was determined by SDS-PAGE [31].

#### Isolation of paralyzing extract

300 mg of the neurotoxic phase was passed through a C18 Solid Phase Extraction cartridge (Xtrata 1 mg/6mL, Phenomenex, previously washed and conditioned with methanol and water, 5 mL each phase), then the paralyzing fraction was eluted by adding 10 mL deionized water. The fraction recovered was lyophilized and tested (100 μL of 25 mg/mL of fraction) in the bioassay.

#### Amino acid profile of paralyzing extract

10 μL of the paralyzing fraction was derivatized according to the AccQ-Tag protocol (Waters Inc. Mass., USA). Amino acid profiles were determined following [29].

#### Isolation of relaxing fraction

After recovery of the paralyzing extract, the cartridge was immediately gradient eluted (95:5, 90:10, 80:20 and 50:50 water:acetonitrile (AcCN) phases, 5 mL). All fractions were recovered and lyophilized. Fraction 2 (F2, 90:10 water: AcCN) induced relaxation and loss of coordination in a ghost crab for 2 h (dose 8 mg/kg).

Fraction 2 was separated in a Strong Cation Exchanger SPE cartridge (Strata SCX, 1 g/6 mL), previously washed with MeOH and conditioned with 10% TFA in water (5 mL each). The sample (50 mg) was diluted with 10% TFA in water and applied to a cartridge, followed by two washes with water, one with 0.1 N HCl in water, and then with 0.1 N HCl in MeOH. Finally, the active fraction was eluted with 2% NH_4_OH in MeOH and vacuum dried (F3, 12 mg). F3 analysis by HPLC in a reverse-phase column (C18, Luna) revealed a single peak, which in turn was injected in a semi-preparative run and manually collected, and lyophilized to obtain 2 mg of white solid. This solid was dissolved in deuterochloroform, and analyzed first by NMR (^1^H and ^13^C) and then by GC-MS.

#### Extraction of saliva *in vivo*

Thirty octopus were collected around the Sisal area as described above. Each octopus was anesthetized in a watery ice bath for 30–40 min and placed in a dissection tray with ice underneath. Animals were euthanized by an incision between the eyes [26]. A dorsal incision was made to the octopus mantle and the esophagus and venosus sinus above the PSGs were dissected. The PSGs were carefully freed from the four arteries that are connected to them. Those are two arteries that connect the PSGs to the anterior stomach (Crop) and two arteries that connect the PSGs to the buccal mass [15]. The common duct was cut and stimulated with a pair of power cables attached to a 9V battery. The secretion was collected in vials and preserved in an ultra-low-temperature freezer at -70°C (REVCO, thermo-Science, USA). This procedure was performed to collect at least 2 mL of saliva in a single vial; this saliva was then separated by the bioguided PSG scheme described above, and fractions were compared with PSG paralyzing and relaxing extract isolated by HPLC profiling.

### Behavior of *Octopus maya* fed with Callinectes sapidus

The interaction between large or small octopus and large or small blue crabs was examined to characterize the feeding behavior of *O*. *maya* and to determine whether prey handling differed according to predator and prey sizes. This approach resulted in four different predator-prey combinations: Large octopus-Large blue crab, Large octopus-Small blue crab, Small octopus-Large blue crab, Small octopus-Small blue crab, with 25 independent replicates each.

Prior to the experiments, octopus were offered fresh crab meat *ad libitum* and were then deprived for 48 h in order to standardize starvation levels. On the third day, an individual *O*. *maya* was placed in a circular fiberglass arena of 2.5 m in diameter, 60 cm deep, and with a tempered glass bottom (10-mm thick), under which a recording video-camera was situated. A second video-camera was located above the arena to obtain a full view of each foraging bout from two opposite angles. Seawater was introduced into the arena by means of a water pump and changed on a daily basis. Salinity, dissolved oxygen and temperature were set as per the maintenance conditions. Octopus were allowed 10 min to habituate to the arena before an individual crab was introduced, with the crab placed as far as possible from the octopus. Recordings lasted 8 min, a period of time that proved to be sufficient for a prey to be attacked and captured. Immediately after the recording ended, the prey was removed and kept frozen at -70°C for subsequent analysis.

A stereoscopic microscope (Leica, EZ4, HD) was used to examine whether the cephalothorax, eyes and joints of all walking appendages of the manipulated prey had signs of drilling. Depending on the place where the injury was found, prey individuals were classified by the presence of injuries in the eye, arthrodial membrane of any appendage or as items without evidence of drilling.

#### Statistical analysis

The presence/absence of the behaviors displayed by *O*. *maya* in the different treatments was analyzed by Non-Metric Dimensional Scaling (NMDS). The reciprocal of the Sorensen index (S_8_) was used as a dissimilarity measure between samples to compare the presence/absence of behaviors [32] used by predators of different size on prey of different size and amongst prey with different types of injuries. A 3D configuration was defined prior to the iterative procedure of NMDS and the magnitude of the stress was used as quality criterion for the final configuration [32]. In order to better visualize the ordination of samples in the resulting 3D configurations, we produced flattened 2D representations of the 3D maps [33] Vectors representing the behaviors and their corresponding correlation (Spearman coefficient) with each NMDS axis were plotted on the final configuration to identify the contribution of behaviors to the sample ordination. All multivariate procedures were performed with PRIMER 6 version 6.1.14 and PERMANOVA+ 1.0.4 (PRIMER-E Ltd.), following specifications given in the manuals [33].

To compare behaviors of different prey and predator size and type of injury, a permutational MANOVA [34] was applied on the dissimilarity matrices of the behavioral data. The underlying experimental design was a three-factor model with “prey size” (two levels: Small and Large), “predator size” (two levels: Small and Large) and “type of injury” (three levels: eye, arthrodial membrane, no evidence of drilling) as the main factors. Only the three main terms were considered relevant, hence they were statistically analyzed. In each case, 999 restricted permutations of residuals under a reduced model were used to obtain the empirical distribution of *pseudo-F* values [35].

#### Radula functionality: predation upon gastropods

In the first part of the experiment, 10 octopus were individually placed in 80-L tanks. All octopus were left without food for 48 h prior to the experiments to synchronize famine levels. Two crown conch snails (*M*. *corona bispinosa*) were then added to each tank. After 24 h, shells were retrieved from the tanks, and were analyzed under the stereoscopic microscope to determine the location of drilling, its size, form and position.

In the second part of the experiment the location at which previous perforations were found was covered with dental cement (Type 3 dental stone Elite Model, Zermack SpA [7, 36]). The newly reinforced snails were then offered to 10 new octopus with the same fasting level as previously described. Considering that octopus were without food for 48 h, if the radula in *O*. *maya* is functional, perforations should be present in conch snails in both the first and second part of the experiments. If a hole was present then its location should have changed in the second part of the experiment.

#### Onset of activity according to the injection site

Juvenile blue crabs were used (*n* = 6, 103 to 130 g TW, 10 mm CW) and were divided into two groups. Each group was injected with 10 mg/kg of the neurotoxic fraction through a short and extra-fine hypodermic syringe (for insulin) with a 6-mm needle (32 M, BD) to simulate the small entrance of the radula in the eye and in the arthrodial membrane, which is accessed from the swimming leg of the crab. After injection, the onset of neurotoxic activity over the crab was determined.

#### Effects of *Octopus maya* saliva on blue crabs

The effects of saliva were qualitatively examined by feeding *O*. *maya* adults with adult blue crabs that were recovered at fixed intervals (10, 20, 30, 40, 50, 60, 120, 180, 300, and 600 s). For each experiment, an octopus was acclimated for one hour in the arena and the adult crab was introduced afterwards. As soon as the octopus attacked the crab, we recorded the time when the prey was carried to the mouth; this was defined as the initial time, following previous studies on *Eledone cirrhosa* [11].

#### Effects of the neurotoxic fraction on *Octopus maya* conspecifics

Subadult octopuses (n = 6, > 300 g weight) were divided into two groups. The first group was injected with isotonic saline solution and the second group with the neurotoxic fraction (intramuscular dose of 20 mg/kg of octopus weight, neurotoxic fraction was 0.2 mg/μL) in the arm. After injection, each organism was observed for at least 8 h and the effects were recorded.

## Results and Discussion

### Chemical studies of *Octopus maya* saliva

The PSGs collected from fishery byproduct were extracted with distilled water and separated by molecular weight through an ultrafiltration membrane (3 kDa MWCO) giving two different fractions: 1) metabolic, MW > 3 kDa, that induced death of the ghost crab after 20 min, and 2) neurotoxic, MW < 3kDa, that induced shaking, trembling and postural changes. The neurotoxic fraction was separated by a reverse phase SPE C18-U technique, leading to a paralyzing fraction and a relaxing fraction (90:10 H_2_O: acetonitrile) (Table 1).

**Table 1:** Saliva from posterior salivary glands of *Octopus maya*; extraction, separated fractions and effective dose 50 (ED_50_) in ghost crab.

Fraction	Total (g)	Yield (%)	Saliva *in vivo* (mg)	ED50 (mg/mL)
Posterior salivary glands (wet weight)	503	100	nd	nd
Posterior salivary glands (dry weight)	110	21.86	nd	nd
Crude extract	40.92	8.14	*	4
Neurotoxic Fraction (< 3kDa)	17.639	3.50	92.4	2
SPE C18-U, paralyzing fraction, 100% water	8.74	1.73	13.6	25
SPE C18 U, F2, Sedating fraction, 90:10, H_2_0:AcCN	1.48	0.29	6.3	1
SCX, F3, Sedating fraction, 2% NH_4_OH in MeOH	0.11	0.02	1.6	0.1

*1800 μL obtained from 34 octopus;

nd, not determined. SPE = Solid phase extraction, SCX = strong cation exchanger.

Yield of crude extract was higher than expected given that the octopus generates saliva *in situ* but PSG tissue was extracted with water; hence salts, carbohydrates and proteins were also extracted and may have been present in the saliva. Nonetheless, the extract was active and able to induce paralysis of the crab after 5 min and death after 20 min (4 mg/mL, 100 μL).

**Fig 1:**
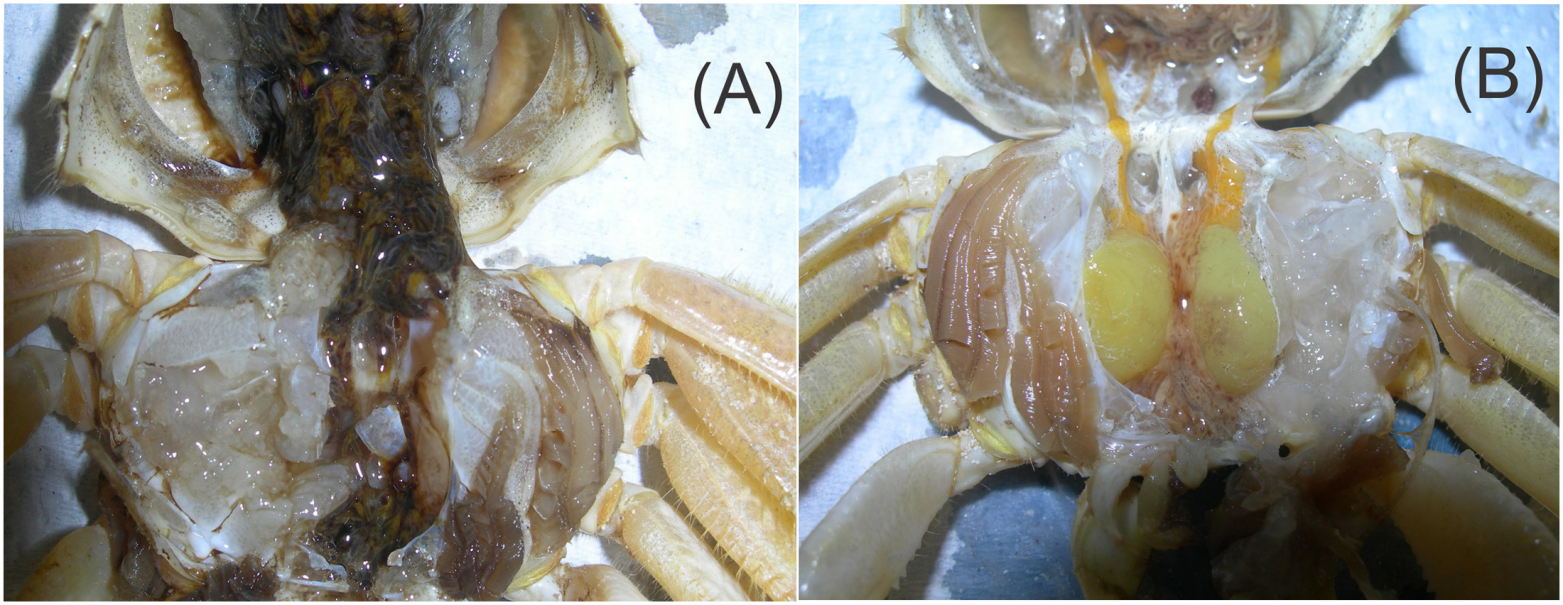
Effects of *Octopus maya* saliva on ghost crab. *Ocypode quadrata*: (A) tissue hydrolysis and death caused by the metabolic fraction (> 3 kDa); (B) organs still intact after the crab had recovered from paralysis and relaxation induced by the neurotoxic fraction (< 3 kDa).

Separation by ultrafiltration (3 kDa MWCO) helped to isolate the metabolic fraction (comprising mainly trypsin and chymotrypsin with MW ~24 and 25kDa, respectively) from the neurotoxic fraction [19], as demonstrated in the bioassay. The neurotoxic fraction induced paralysis, muscle relaxation and postural changes in the ghost crab in less than one minute. The neurotoxic effects lasted 2 h followed by recovery of the crab. The metabolic fraction caused death after 20 min but there was no evidence of neurotoxic effects; dissection after 1 h of these crabs revealed damage exerted by the proteolytic enzymes in the metabolic phase (Fig 1A), whereas this was not seen with the neurotoxic fraction.

#### Paralyzing fraction

Elution with water of the neurotoxic fraction applied in a C18-U SPE cartridge resulted in a fraction that caused total paralysis of the crab. However, doses of the paralyzing fraction (25 mg) were higher than those of the neurotoxic fraction (4 mg) (S3 video). Thin-layer chromatography in normal phase showed that the neurotoxic fraction was polar with the compounds revealed with ninhydrin, which suggested a peptide or primary amine origin. HPLC in reverse phase using ELSD detector showed two peaks in the 3 to 4.8 min range (S1 Fig), as expected since compounds were eluted from the SPE cartridge with water.

Analysis revealed 17 amino acids in the paralyzing fraction (S2 Fig), including alanine and glutamine, which occur in the central nervous system of crabs and lobsters [37]. Some of these amino acids are involved in muscular inhibition, e.g. glutamic acid, which can be transformed into gamma-aminobutyric acid, an inhibitor of the moto-neuron function in pereiopods of crabs [38].

#### Relaxing fraction

Subsequent to the separation of the paralyzing fraction, the C18-U cartridge was gradient eluted with water-acetonitrile (0–50%). All the fractions were evaluated in the neurotoxic assay; only fractions 95:5 (low activity) and 90:10 (F2, 2 mg/mL, 100 μL) caused relaxation in the ghost crab. Both fractions induced reversible relaxation, and the crab recovered and had no symptoms after two hours. Two days later, no crab had died.

Separation of the relaxing fraction (F2) with the SCX-SPE cartridge led to a fraction with strong activity on the crab (F3, 2% NH_4_OH in MeOH, dose of 1 mg/mL, 100 μL); the crab was relaxed after 10 s (S4 video). HPLC reverse-phase analysis revealed a major single peak, which in turn was separated by a semi-preparative HPLC run (5 mg / 25 μL) and the principal peak collected. Structural analysis (S3 Fig) indicated that serotonin was isolated: ^1^H NMR (CDCl_3_, 400 MHz, δH) 3.13 (2H, t, CH_2_-CH_2_-NH_2_), 3.33 (2H, t, CH_2_-CH_2_-NH_2_), 7.12 (1H, d J = 2.4 Hz, H-6), 7.43 (1H, d, J = 8.7 Hz, H-4), 7.89 (1H, dd, J = 8.7 Hz, H-5); ^13^C NMR (CDCl_3_, 400 MHz, δC) 22.6 (CH_2_-CH_2_-NH_2_), 39.6 (CH_2_-CH_2_-NH_2_), 102.5 (C-7), 108.4 (C-2), 111.9 (C-5), 112.9 (C-4), 125.3 (C-1), 127.6 (C-8), 131.6 (C-3), 148 (C-6); GC-MS (Direct insertion, the sample was deuterated because it was injected after NMR studies, deuterated MW calculated: 179.1, found [M+]:179.1, 149.1 [M-CH2-NH2+]), 2D-NMR experiments validated the structure (S4 Fig). We confirmed the identity of serotonin by injecting a serotonin standard (2 mg/mL, Sigma Aldrich H9523) in the HPLC system. The retention time was identical to the peak isolated in the F3 fraction. Moreover, evaluation of serotonin in the neurotoxic bioassay (2 mg/mL) induced relaxation in the crab with the same time and intensity.

Serotonin is a biogenic amine that has been isolated from the venom of other octopods, e.g. *Hapalochlaena maculosa* and *O*. *vulgaris* [39, 40]. Like many other monoamines, serotonin may cause immobilizing hyper-excitation that precedes paralysis or hypo-kinesia of prey [41]. In addition, serotonin usually circulates in free form in crustaceans, e.g. a concentration of 1–8 nM is found in lobsters. Injections of serotonin induce postural changes in crabs, mainly flexion and extension of the walking legs and chelae, both in *C*. *sapidus* and in *Carcinus maenas* [42]. Postural changes and time effects are dose dependent, e.g. low doses of serotonin (0.5 to 1.0 mg) induce rigid flexion of legs and abdomen for 10 to 30 min in the crayfish *Procambarus clarkii*, whereas extreme flexion that lasts several hours is observed if larger doses (1.0 to 10 mg) are injected [42]. Moreover, some postural changes are conspicuous (S5 Fig) as previously reported [43], for instance, the “cradle carry” position exhibited by blue crab males during mating.

#### Saliva *in vivo*

The presence of amino acids in the crude extract remained unclear because the fraction was obtained by water extraction with dead tissue, and the degradation and the autolysis of dead tissue may have produced free amino acids. The volume of saliva that *O*. *maya* uses to subdue its prey was determined, and the HPLC profile and yield were compared with the PSG extract from the fishery byproduct.

Early attempts to obtain saliva by an envenomation technique [44] were unsuccessful because of the elusive nature of *O*. *maya*. However, envenomation worked well with aggressive octopus such as *Eledone* spp. Saliva was collected from a live *O*. *maya* that was dissected less than 1 minute after sedation. The PSG and common conduct were stimulated with a 9 mV battery to obtain the saliva *in vivo* (less than 10 min passed from the dissection to the collection of the saliva). *In vivo* saliva was separated according to the PSG scheme; results are presented in Table 1.

**Fig 2:**
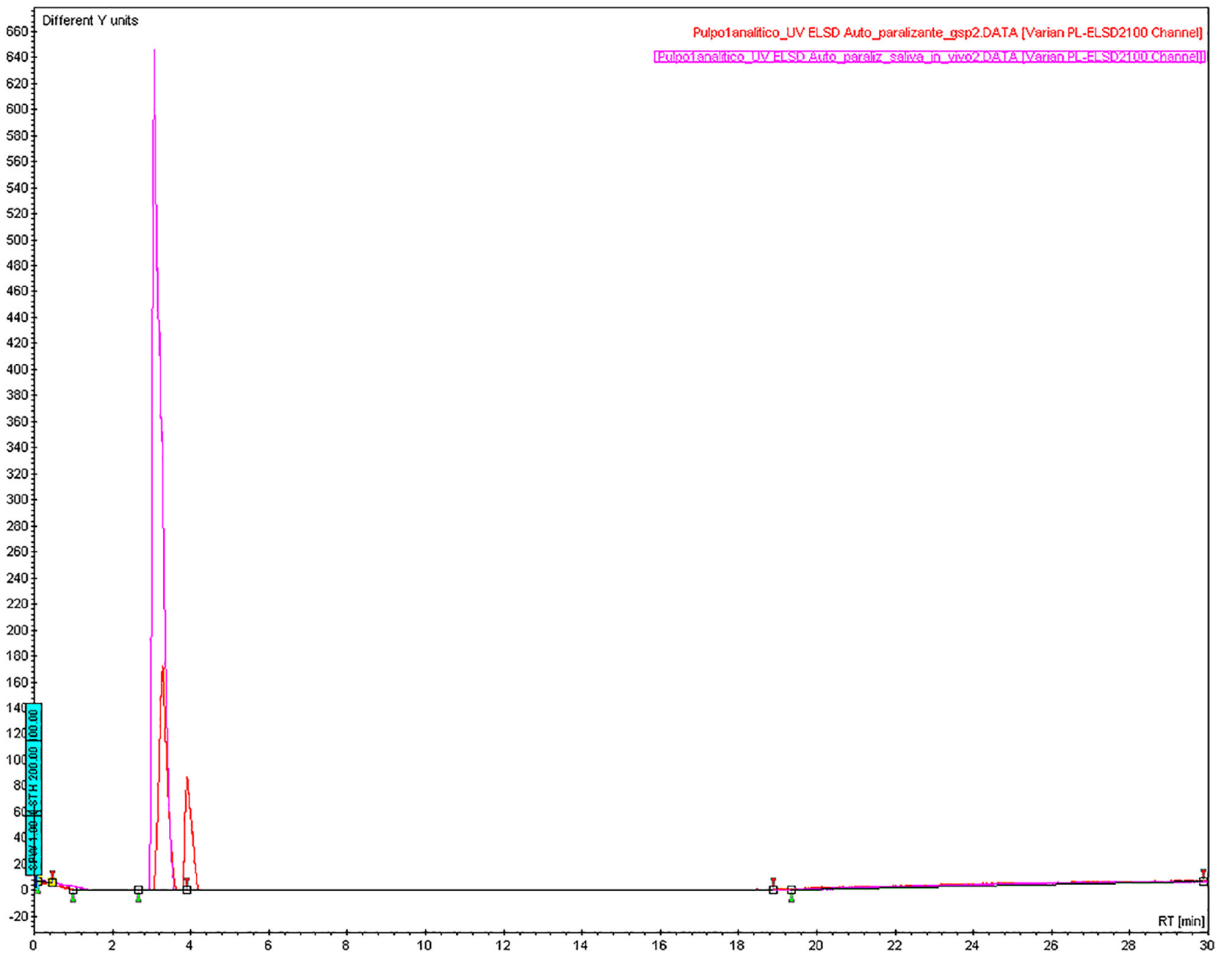
Comparison of paralyzing fractions of *Octopus maya* saliva. The HPLC chromatogram from C18 reverse-phase separation of the paralyzing fraction from posterior salivary glands (red) provided by fishers matched the paralyzing fraction from saliva *in vivo* (pink). Detection: ELSD, neb. temp = 40°C, evap. temp = 80°C, flow 1 mL/min.

**Fig 3:**
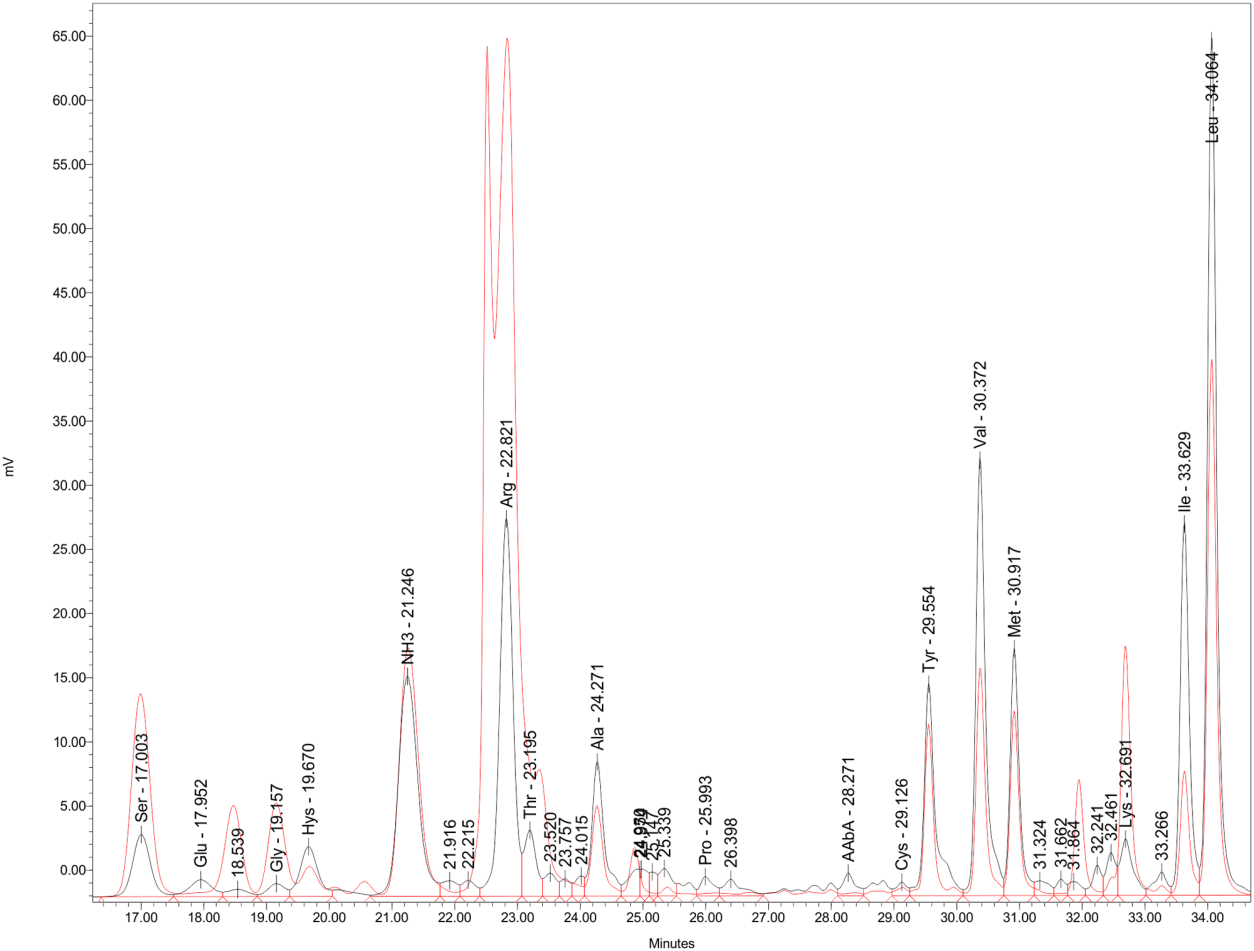
Comparison of amino acid profiles of the paralyzing fractions of *Octopus maya*. Amino acid profile of the paralyzing fraction from posterior salivary glands (PSG; pink) provided by fishers matched the paralyzing fraction from saliva *in vivo* (black). Eleven of the amino acids from the saliva are present in PSG aminogram in nearly the same amounts. Detection with Waters 2470 fluorescence detector, conditions: excitation wavelength: 250 nm, emission wavelength 395 nm [29].

The fractions obtained presented nearly the same bioactivity in the neurotoxic bioassay. Thin-layer chromatography of the paralyzing fraction, using ninhydrin (2% in n-butanol), revealed only one spot. The first peak (tr = 3.2 min) detected by the HPLC chromatogram matched the first peak in the PSG extract (Fig 2). The amino acid profile (Fig 3) of the PSG extract *in vivo* detected 11 of the amino acids in nearly the same concentrations found in the PSG extract from the fishery byproducts.

The amino acids in the saliva may be related to metabolic requirements and may support energetic processes and growth of cephalopods, including *O*. *maya*. A high demand for protein requires a high intake of amino acids to be absorbed in the octopus stomach, and an active transport [29].

### Behavior of *Octopus maya* fed with Callinectes sapidus

The behavior of *O*. *maya* (Table 2) appeared to depend on prey size, with the octopus adopting three different attack strategies:

**Table 2:** Predatory behaviors displayed by *Octopus maya* offered crabs (*Callinectes sapidus)* during foraging experiments under laboratory conditions.

Behavior Type	Behavior name	Description
Detection	‘Mov’	Crab was moving at detection time
Detection	‘Detal’	Octopus adopts an alert position but does not change color.
Detection	‘Detco’	Octopus changes color but does not change body position.
Detection	‘Detmi’	Octopus changes color to mimic the background but does not change body position.
Attack	‘Ataq’	Octopus ambushes the crab and does not move to another location within the arena.
Attack	‘PosP’	Octopus faces the crab and extends the first pair of arms forward to make contact.
Attack	‘PosJ’	Crab receives attack facing the octopus with chelae spread out in a defensive position.
Attack	‘Prop1’	Rapid propulsion by octopus towards the crab during the attack.
Attack	‘Prop2’	Slow propulsion by octopus towards the crab during attack.
Attack	‘Inm’	Octopus seizes the crab, completely impeding its movement by holding both chelae with the arms before embracing the crab under the inter-branchial membrane.
Manipulation	‘Col1’	Octopus positions the crab to face its anterior side (rostrum, mouthparts).
Manipulation	‘Col2’	Octopus positions the crab to face its posterior side (last pair of pereiopods)
Manipulation	‘Col3’	Octopus positions the crab to face either side (other walking legs).
Manipulation	‘Desp’	Octopus moves within the arena without letting the crab go.

1. Frontal—The octopus confronted the crab by facing its chelae, which were held, immobilized and forced to a ventral position with respect to the buccal mass of the octopus. The octopus appeared to use this technique to minimize damage, since chelae of the crab remained near and might have caused injury to the predator; only large *O*. *maya* presented this strategy (S5 video).
2. Posterior—This consisted of an ambush attack where the predator was out of reach of the prey chelae. In addition, the octopus remained out of the visual field of the crab, which prevented the crab from taking evasive action. Both large and small octopus used this strategy (S6 video).
3. Lateral—The octopus attacked the prey from the side. This allowed the predator to occupy a larger flank to attack the prey, whose position was no longer important. The octopus opted for this strategy especially when the crab was small or when the crab chelae were large (S7 video).

Overall, crabs were detected by octopus upon movement (Mov, Table 2, S1 Table); only 0.04% of crabs were motionless when detected by the octopus. This indicates that visual cues from the prey were the most important for detection. This agrees with other reports that some cephalopods are visual hunters guided by the movements of their prey [4, 14, 45]. Following detection of the prey, 75% of the octopus changed color (Detco; Table 2) and almost half did so to mimic the background of the arena (Detmi; Table 2). Similar results have been previously reported in octopods [45, 46]; the changes in color during foraging might be advantageous, particularly when corporal patterns can make them imperceptible. The octopus attacked either by ambush (46%) or by fast movements in pursuit of the crab (54%), with the predator either facing the prey with the first two arms extended forward or approaching the prey sideways (Ataq, PosP, Table 2). In 35% of trials, blue crabs received octopus attacks with both chelae spread apart, open in a clear defense position; since the prey was completely immobilized by octopus in only 27% of all foraging bouts, this defense position might give crabs an advantage. In all other trials, crabs were still capable of slightly moving the walking appendages and chelae, thereby retaining the ability to inflict damage to the octopus arms. Once crabs were captured, they were drawn towards the underside of the octopus from the front, from the rear, or sideways (Col1, Col2, Col3; Table 2). From the moment crabs were entirely enveloped by the web, crabs were left with both chelae completely unharmed. In at least 33% of the tests, the octopus moved to another place in the arena carrying the recently captured prey. This behavior suggests that in its natural environment the octopus seeks shelter in its den immediately after capturing a prey.

**Fig 4:**
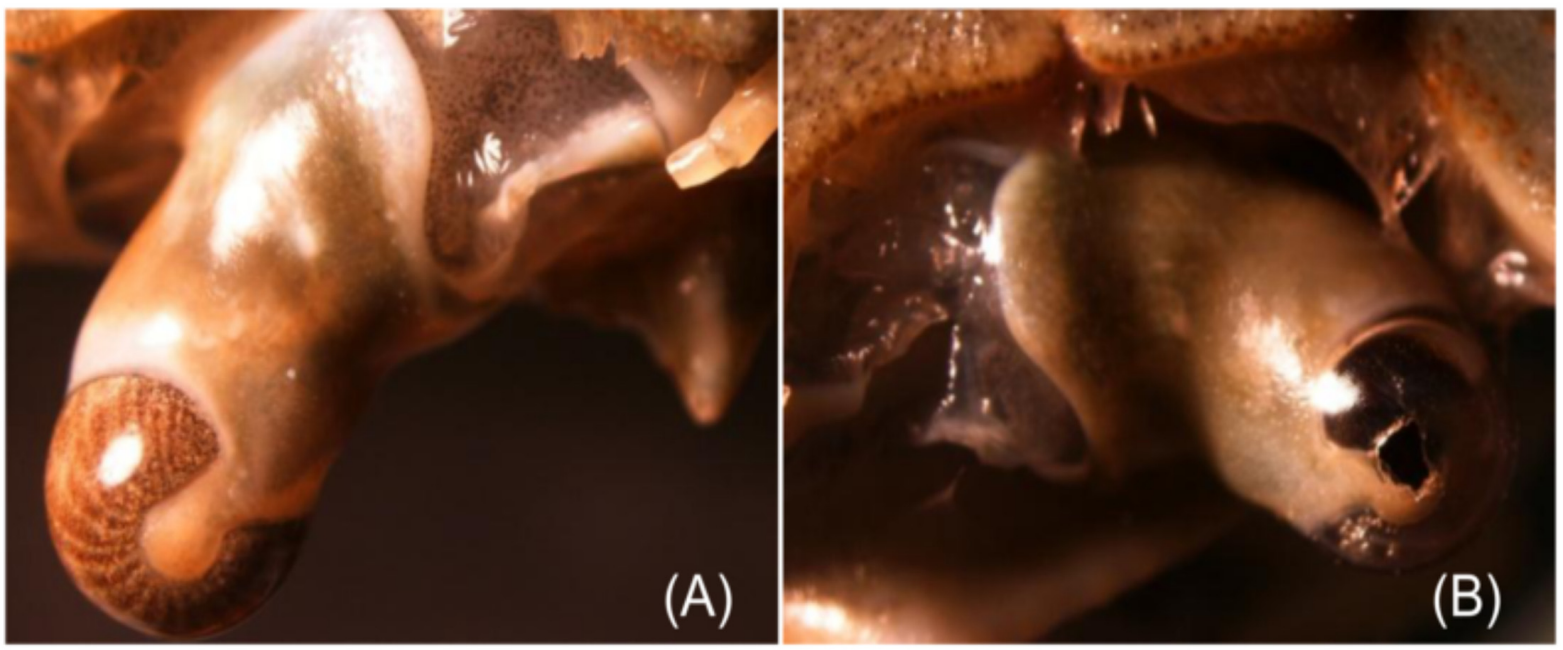
Injury to the eye of *Callinectes sapidus* after an attack by *Octopus maya*. A) Normal physiognomy of the peduncle eye (stereoscopic microscope 3X). B) Injury to the rostrum of a crab attacked by *O*. *maya* (stereoscopic microscope 3X).

**Fig 5:**
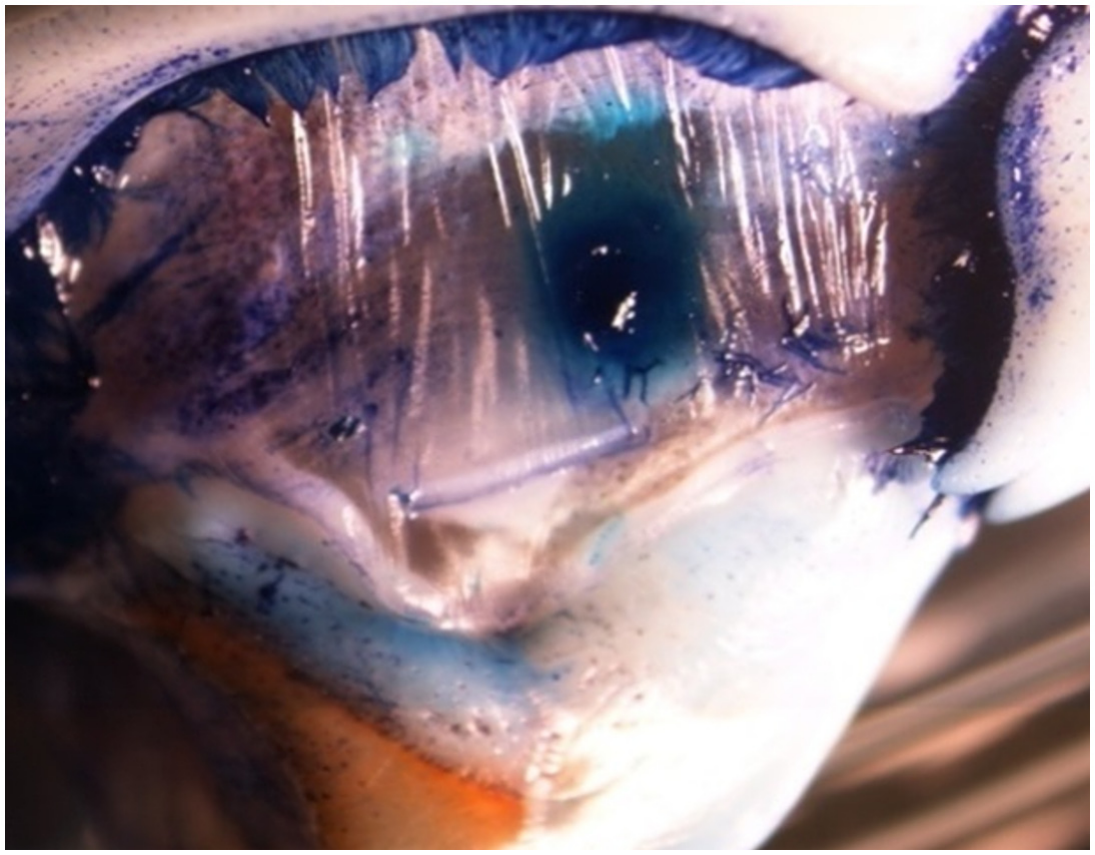
Injury to the arthrodial membrane of the coxa and swimming leg of *Callinectes sapidus* after an attack by *Octopus maya*. The prey was attacked from the rear side. (Methyl blue dye, stereoscopic microscope 3X).

All the foraging bouts (n = 100) ended with the successful capture of crabs irrespective of crab and octopus size. However, none of these bouts showed evidence of drilling on the crab carapace. Whilst eight crabs were overcome by puncturing the orbital eye (Fig 4), 22 crabs were overcome by puncturing the arthrodial membrane of the last pair of walking pereiopods. Unfortunately, it was not possible to determine whether the damage was inflicted by the radula or the beak (Fig 5). No evidence of perforations was found in the remaining 70 crabs captured. The lack of drilling on the carapace raises questions regarding the role of the radula when *O*. *maya* predates on crabs.

**Fig 6:**
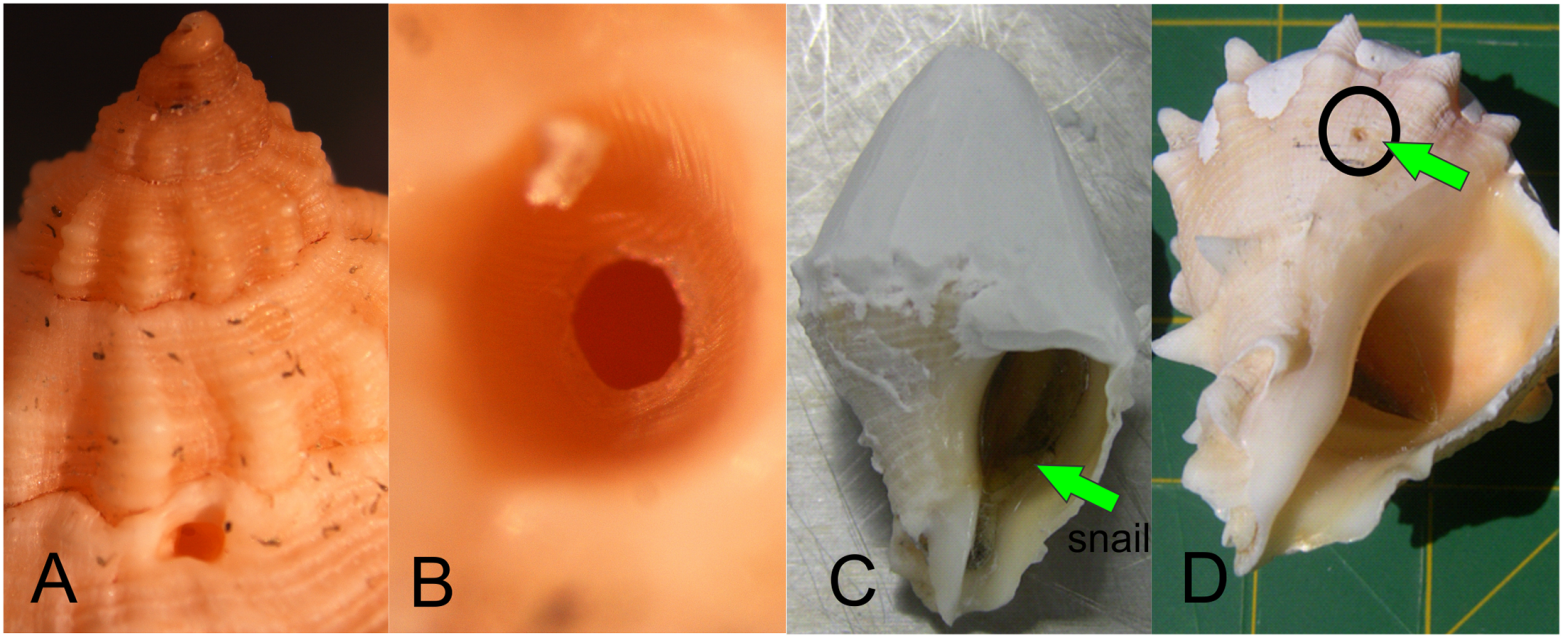
Drilling on the shell of *Melongena corona bispinosa* by *Octopus maya*. A) Hole below the apex in the fifth loop suture. B) Dissolution and scraping at the edge of the hole. C) Conch shell coated with dental cement over the posterior end. D) Drilling below the line of the dental cement, and closer to the inner lip of the conch.

Octopus feeding strategies changed when they were offered the Crown conches. *O*. *maya* drilled the shell of every snail in the same place, just below the apex in the fourth or fifth suture of the shell (Fig 6A). This confirms the role of the radula in *O*. *maya* when preying on gastropods. Gastropods under risk of predation retract inside the shell and close the opening with the operculum; by drilling, the octopus gained access to the visceral mass of the snail. The configuration of the resultant holes suggested the physical action of the radula (scraping, Fig 6B) and chemical action of the saliva (dissolution, Fig 6B). Our results support the evidence that the radula is active [7, 16, 47, 48].

When octopus were offered a conch whose shell had been partially coated with dental cement to cover a previously drilled hole, they found the limit of the coating (Fig 6C) and drilled a new hole (Fig 6D). Although some octopods can drill both layers (e.g. *O*. *vulgaris* [6]), *O*. *maya* appears to use an easier strategy to prey upon gastropods. For instance, *O*. *maya* mostly uses its radula when the prey has hard external structures for protection. At the time of injection of the saliva, the secretion is generated in two PSGs joined by the common duct and discharged through the salivary papilla that allows it to flow into the hole and disperse through the radula. The radula is the main hard structure that octopods use for drilling, although the papilla and its front are covered with very small teeth that can function as an accessory radula [48, 49]. However, we suggest that *O*. *maya* may only use the salivary papilla for injection of saliva into the hole drilled on the prey. An exhaustive search using a stereoscopic microscope failed to find dentition of the salivary papilla, but additional approaches are needed to determine whether such dentition is present, e.g. scanning electron microscopy.

*Octopus maya* may be using the most efficient strategy to subdue its prey, since despite the functionality of the radula no drilling was observed in the predation of blue crabs. When octopus injected saliva into the arthrodial or eye puncture, the crab was paralyzed in less than one minute, a strategy that takes less time than drilling [47, 50]. If the risk of exposure while hunting is high, octopus may maximize efficiency not by optimizing energy gain but by reducing their own vulnerability to predation [4, 51]. According to our results, *O*. *maya* may be classified as a time-minimizing hunter since it appears to have similar hunting traits to those of *Enteroctopus dofleini* and *O*. *insularis* [4, 51].

**Fig 7:**
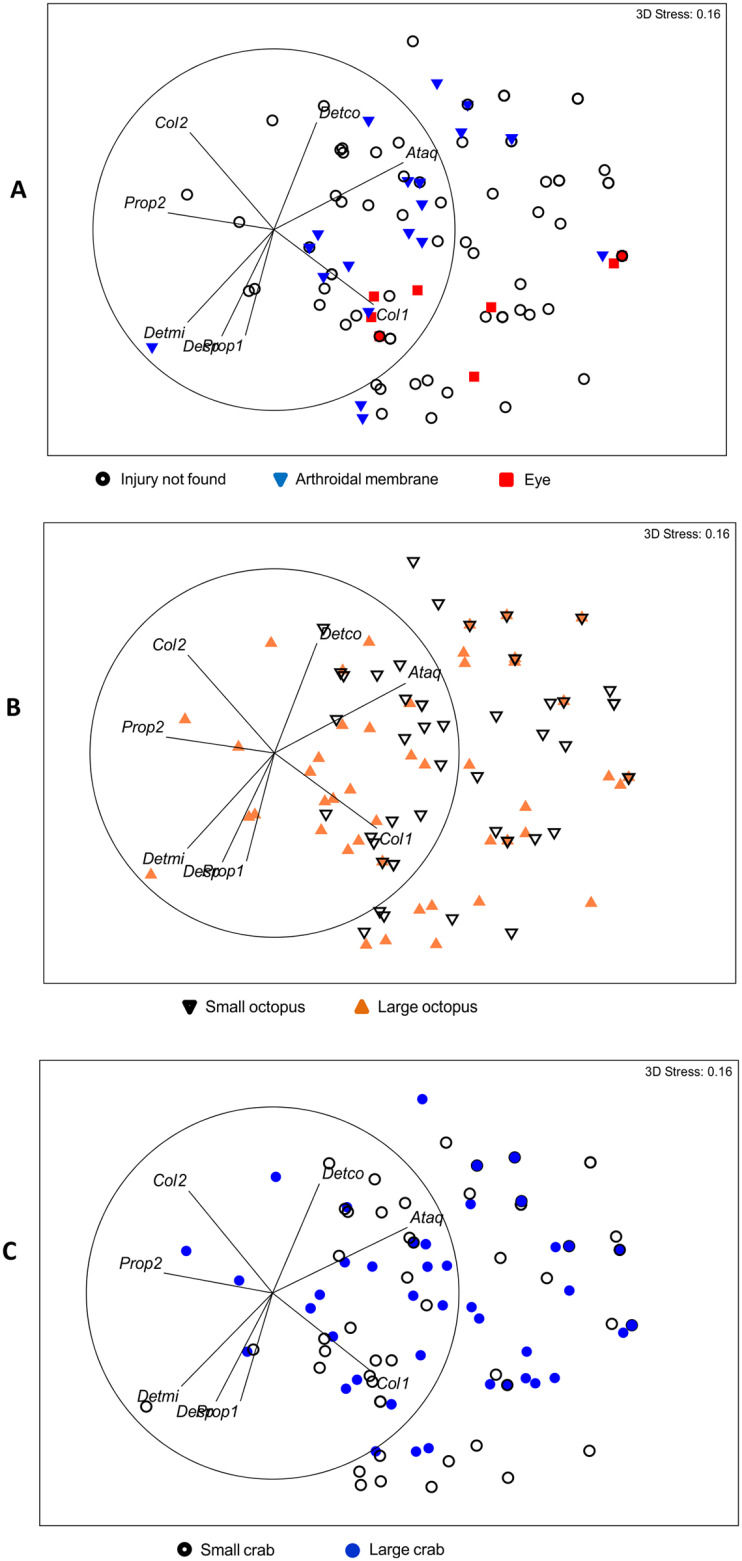
Final 3-Dimension configurations of behaviors of *Octopus maya* when a blue crab (*Callinectes sapidus*) was offered as prey. (A) Position of injury, (B) sizes of predator, (C) sizes of prey. NMDS analysis; vectors representing the behaviors (see Table 2) and their corresponding correlation (Spearman coefficient) with each NMDS axis were plotted on the final configuration to identify the contribution of behaviors to sample ordination.

The 3D ordination of samples obtained through NMDS had a stress of 0.16, indicating a “sufficiently good configuration that requires caution” in graphic interpretation [35]. The configuration showed a clear separation of samples between blue crabs that had been injured in the eye and those injured in the arthrodial membrane of the last walking appendage (Fig 7A). This separation appears to correlate well with the position in which crabs were placed and drawn towards the octopus just before they were enveloped by the web, as indicated by the close correspondence between samples with eye and membrane injury with vectors Col1 and Col2, respectively (Fig 7A and Table 2). These results, together with those of the permutational MANOVA (Table 3), indicate that the type of injury inflicted on crabs depends on the position in which crabs are drawn under the interbranchial membrane during the last moments of the attack.

**Table 3:** Results of permutational MANOVA on behavioral data (presence/absence) displayed by *Octopus maya* when blue crabs *Callinectes sapidus* were offered.

Source	df	SS	MS	Pseudo-*F*	Probability (perm)	Unique permutations
Octopus size	1	6115.2	6115.2	4.35	0.003	998
Crab size	1	5233.2	5233.2	3.72	0.011	997
Type of injury	2	12755	6377.4	4.54	0.001	998
Residuals	95	1.34E+05	1405.6			
Total	99	1.58E+05				

Terms for the main treatments were: octopus size (large, small); crab size (large, small); type of injury (eye, arthrodial membrane, undetermined).

Most samples representing large octopus were associated with vectors Prop1, Detmi and Desp, whereas those representing small octopus were associated with vectors Detco and Ataq (Fig 7B). This suggests that large octopus more frequently changed color to mimic the background and propelled rapidly towards crabs during attack (Prop1, Detmi; Fig 7B); they also often moved within the arena once the crab had been captured (Desp; Fig 7B). Small octopus, by contrast, changed color but did not mimic the background during attack (Detco), and they used ambush more frequently than larger ones (Ataq, Fig 7B).

Regarding crab size, vector Prop2 and Col2 were behavioral descriptors associated with large crabs (Fig 7C), suggesting that *O*. *maya* slowly propelled towards a large crab during attack and most prey in this size class were manipulated so that their posterior parts were towards the octopus. Small crabs were more often manipulated so that their mouthparts and rostrum were facing the octopus (Col1). Results of the permutational MANOVA confirm these descriptions since all main terms in the model contributed significantly to the ordination of samples based on the 14 behavioral variables analyzed (Table 3).

#### Onset of effect according to the injection site

The neurotoxic fraction was injected in both the arthrodial membrane and the eye of the blue crab, in order to determine the fastest route used by the octopus to paralyze the crab. Although the mechanisms of injection of *O*. *maya* involve a fine flow of saliva through a punctured surface and it is spread with the radula, injection with the insulin syringe was considered a sufficient simulation. Our results suggest that the arthrodial membrane is a more common puncture site than the eye. Indeed, injection on each arthrodial membrane in the pereiopods showed six potential sites on the swimming leg that led to neurotoxic effects in less than 20 s. Furthermore, accessibility of the site favored arthrodial injection, since the eye is protected by the ocular orbit. Injection on the eye also paralyzed the prey in exactly the same time as the arthrodial injection, an expected result because the chemical content of the saliva reaches the hemolymph of the crab and consequently the central nervous system.

#### Effect of the saliva of *Octopus maya* on the blue crab

The prey experienced a gradual paralysis after saliva injection by octopus. Paralysis was irreversible after 3 min of injection and was followed by death as previously reported [11]. The neurotoxic effect of saliva after different time intervals (Table 4) progressed through (1) loss of coordination, (2) immediate contraction or trembling of appendages, and (3) spasms in the rear appendages [15]. These symptoms caused a gradual paralysis during the first minute, with the spontaneous movements disappearing two minutes later and the aggressive prey becoming an inert body.

**Table 4:** Effects of the saliva of *Octopus maya* at increasing time intervals after injection into the blue crab.

	Effects on blue crab							
Time (s)^a^		Pereiopods						
	Loss of coordination	reaction	contraction	trembling	Spasms	Loss of spontaneous movements	Paralysis	Death
10	1	0	0	0	0	0	0	0
20	1	1	0	0	0	0	0	0
30	1	1	1	1	0	0	0	0
40	1	1	0	0	1	0	0	0
50	0	0	1	1	0	0	0	0
60	0	1	1	1	0	0	0	0
120	0	0	0	0	0	1	1	0
180	0	0	0	0	0	0	0	1
300	0	0	0	0	0	0	0	1
600	0	0	0	0	0	0	0	1

^**a**^ Fixed time to recover the crab after the octopus injected the saliva.

The rapid injection of saliva of *O*. *maya* and its quick neurotoxic effect on the prey facilitates the retention of the crab under the web and avoids damage to the octopus by the cutting chelae. In addition, such effects also reduce the need for manipulation by *O*. *maya* of the inactive prey and facilitates predigestion. Comparing these results with the paralysis induced by the neurotoxic fraction isolated (F3) revealed no differences in the onset of paralysis, less than 10 s. However, the exact time when the octopus injects saliva remains an enigma; the octopus envelops its prey so tightly with its inter-arm webbing that this event is almost impossible to detect even with the use of ultrasound scanning [11].

#### Effects of the neurotoxic fraction on *Octopus maya* conspecifics

**Fig 8:**
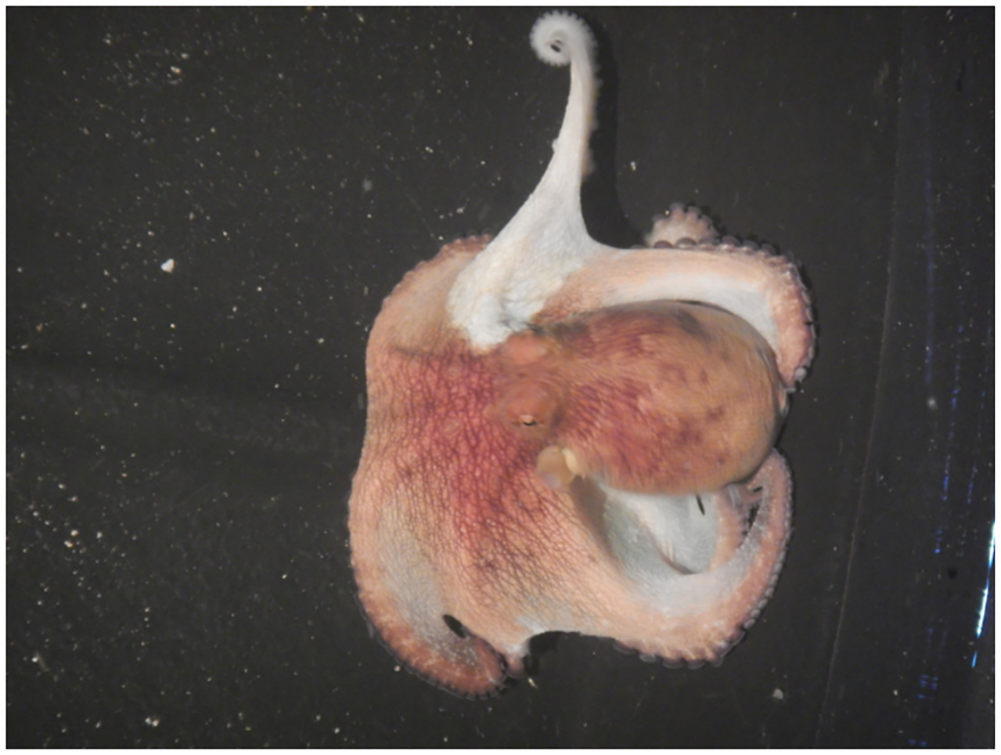
Effect of the relaxing fraction (F3) injected in the arm of an *Octopus maya* conspecific. The injected arm is paling, an effect caused by serotonin in the saliva.

The relaxing fraction (F3) had effects on *O*. *maya* conspecifics similar to those on crabs: body relaxation (5 min after injection) with color change (from red to white), decrease in aggressiveness, and paralysis at the injection site (40 min) without responses of chromatophores; clear signs of sedation on injected areas (Fig 8). Octopus recovered mobility and some chromatophores started to respond after 3 h, and the initial state was recovered after 5 h. Paling is an indication of the effect on chromatophores, which are innervated directly from the sub-oesophageal lobes of the brain [18], although it is not clear whether the innervation is centrally or peripherally mediated. The reversibility of the effect is probably attributable to action on specific receptors.

The neurotoxic fraction was injected to determine whether the saliva can paralyze *O*. *maya* conspecifics without killing them; moreover, metabolic and neurotoxic activity may overlap. Cephalopod saliva contains biogenic amines such as serotonin, as well as neuropeptides with effects on the central and enteric nervous systems, e.g. memory, control of motor system, aggression and behavioral patterns [52]. We found that the most conspicuous molecule in the neurotoxic fraction was serotonin. Effects of serotonin on octopods have only been reported in *O*. *vulgaris* injected with a dose of 1–10 mg that caused a mottling color change [53].

In invertebrates, serotonin is involved in motor pattern generation, escape and social status [39]. Some venoms have evolved to manipulate the monoaminergic system, such as the serotonin in *O*. *maya*, via a variety of cellular mechanisms for both offensive and defensive purposes. An early attempt to demonstrate the activity of saliva and tetrodotoxin in conspecifics of the blue ring octopus *Hapalochlaena maculosa* was unsuccessful, although the extract generated from PSG was active over crabs [54]. Here we demonstrated that saliva is not only used as a tool for food supply but also may be used as defense and to minimize competition among conspecifics. The effects observed require the use of a high concentration of saliva and therefore of serotonin with several orders of magnitude above the concentration normally present in the saliva (50 μg/injection of saliva per octopus vs 200 μg/injection GSP). However, *O*. *maya* injects saliva into its prey several times, and hence the levels of serotonin inserted into the prey are likely to be high. In addition, octopus may inject the saliva at specific sites, where it would locally reach high concentrations but not necessarily through the circulatory system [55].

The Directive 2010/63/EU that regulates studies on live cephalopods emphasizes that anesthesia must be used to avoid cephalopod suffering [26]; however an appropriate anesthetic has not been developed. Therefore, ongoing research in our laboratory is examining whether serotonin causes sedation in other octopuses and whether it can be used as a general anesthetic in cephalopods.

## Conclusions

The PSG crude extract obtained was active over many species of crabs. The PSG aqueous extract presented two main activities: a metabolic effect exerted by molecules of > 3 kDa (probably trypsin and chymotrypsin), and a neurotoxic action (molecules < 3kDa) with paralysis and body relaxation. The bioguided separation of the neurotoxic fraction yields a paralyzing fraction with free amino acids as the principal components, whereas a relaxing fraction acts through serotonin. Interestingly, free amino acids were integrated into the feeding behavior. The neurotoxic fraction was active on *O*. *maya* conspecifics, leaving octopus as prey defenseless against predators and corroborating its likely importance in relation to cannibalistic events. The feeding behavior of *O*. *maya* when offered crabs and crown conchs is similar to that observed in other cephalopods. Although the radula was present and active, there was no evidence of its use to drill into crabs. Given that hole-boring is a time-consuming task, its absence here suggests that *O*. *maya* uses a time-minimizing strategy when hunting for this type of prey.

*Octopus maya* is abundant on the Yucatan Peninsula and its diet primarily consists of several species of crabs that are also abundant in that area. Although octopuses have developed strategies to prey upon mollusks, these strategies may not be used as frequently as expected on less abundant gastropods. The paralyzing effect of salivary fluids suggests that octopuses are adapted to predation on crabs with defensive structures.

## Supporting Information

S1 Fig High Performance Liquid Chromatography (HPLC) of the paralyzing fraction. Analysis of the paralyzing fraction using reverse phase HPLC.
(TIF)

S2 Fig Amino acid profile of the paralyzing fraction. (TIF)

S3 Fig ^1^H and ^13^C NMR spectra of isolated compound from the F3 fraction: serotonin. (TIF)

S4 Fig 2D NMR experiments to validate the structure of serotonin. (TIF)

S5 Fig Postural changes of the blue crab *Callinectes sapidus* induced by the fraction F3. (TIF)

S1 Table Absence/Presence data obtained in the studies of predatory behaviors displayed by *Octopus maya* offered crabs *Callinectes sapidus* during foraging experiments under laboratory conditions. (DOCX)

S1 Powerpoint Posterior salivary glands were collected as byproduct of the octopus fishery near Sisal, Yucatán, México. (PPTX)

S1 Video “Jimba y Gareteo”, an ancient traditional fishing art for *Octopus maya*. (MP4)

S2 Video Activity of the neurotoxic fraction on different crabs. Injection of 100 μL (4 mg/mL) into the blue crab (*Callinectes sapidus*), ghost crab (*Ocypode quadrata*) and fiddler crab (*Uca* sp.).
(MP4)

S3 Video Activity of the paralyzing fraction on the ghost crab *Ocypode quadrata*. (MP4)

S4 Video Activity of the relaxing fraction (F3) on the ghost crab *Ocypode quadrata*. (MP4)

S5 Video Frontal predatory attack by *Octopus maya* on the blue crab *Callinectes sapidus*. (MP4)

S6 Video Posterior predatory attack by *Octopus maya* on the blue crab *Callinectes sapidus*. (MP4)

S7 Video Lateral predatory attack by *Octopus maya* on the blue crab *Callinectes sapidus*. (MP4)
