# Supplementary figures and images for: Chemical Tools of Octopus maya during Crab Predation Are Also Active on Conspecifics
Source: PLoS One. 2016 Feb 19;11(2):e0148922. doi: 10.1371/journal.pone.0148922 (PMC4760938; doi:10.1371/journal.pone.0148922)

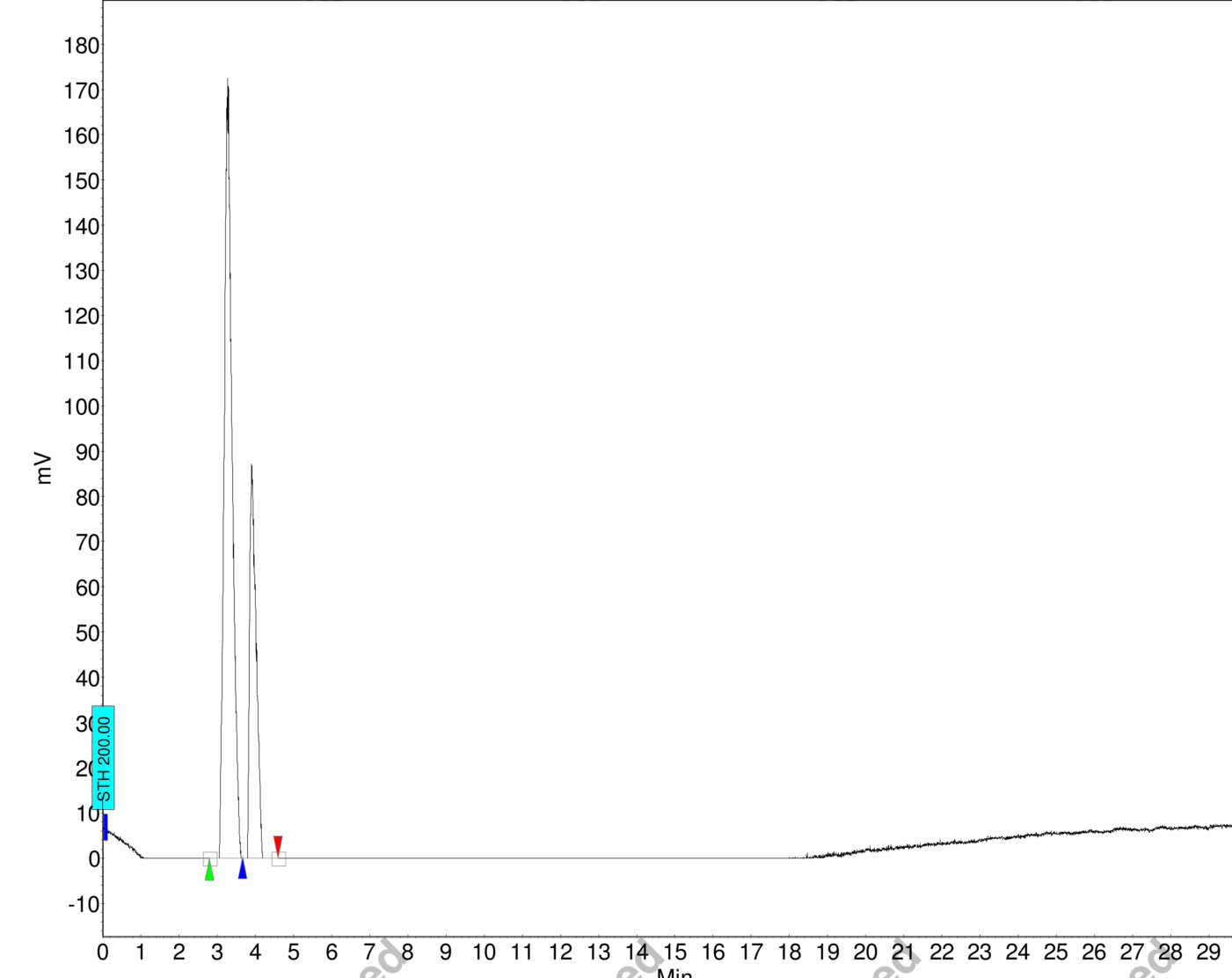

Supplement: S1 Fig — Analysis of the paralyzing fraction using reverse phase HPLC. (TIF) [file pone.0148922.s001.tif]

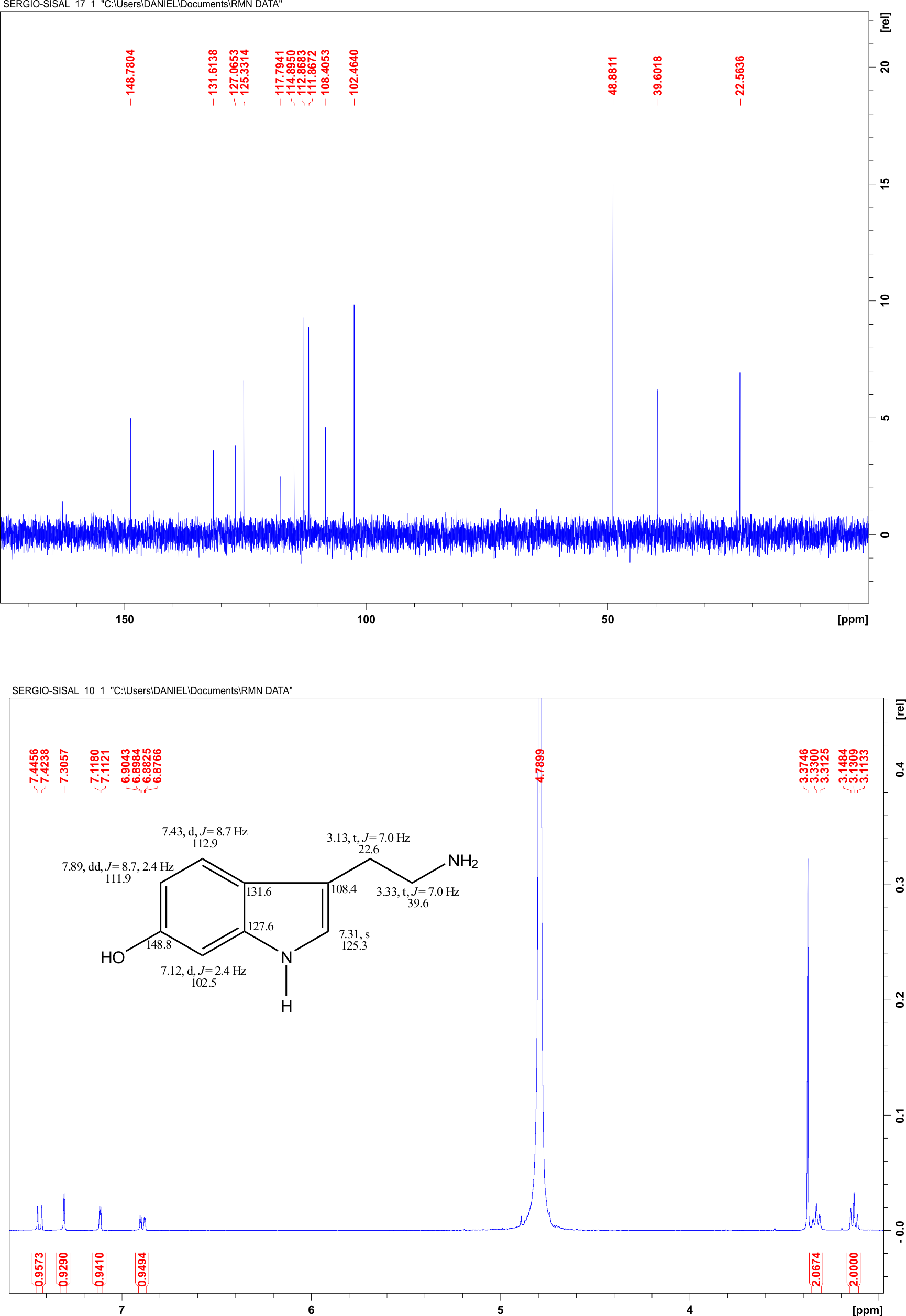

Supplement: S3 Fig — (TIF) [file pone.0148922.s003.tif]

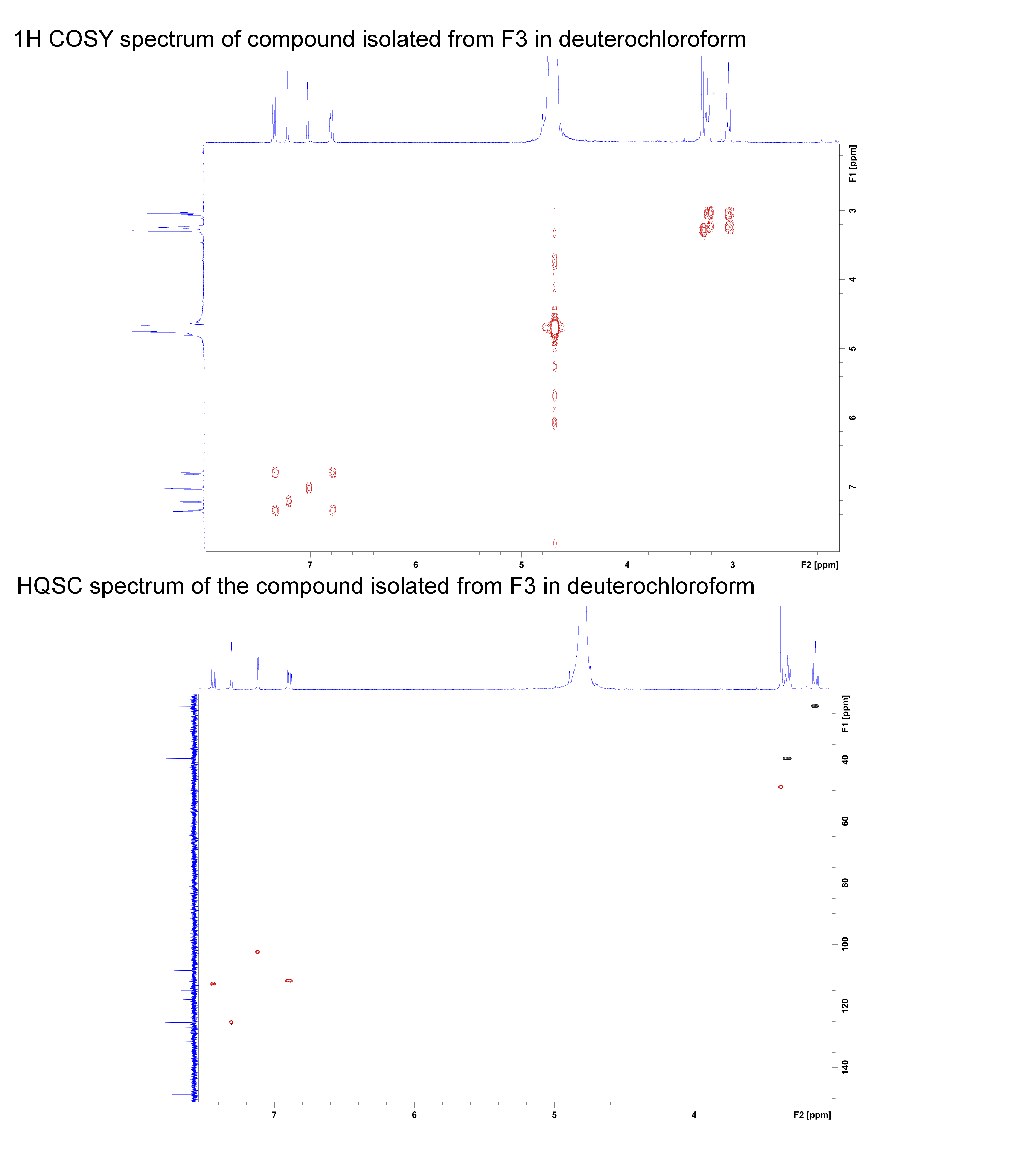

Supplement: S4 Fig — (TIF) [file pone.0148922.s004.tif]

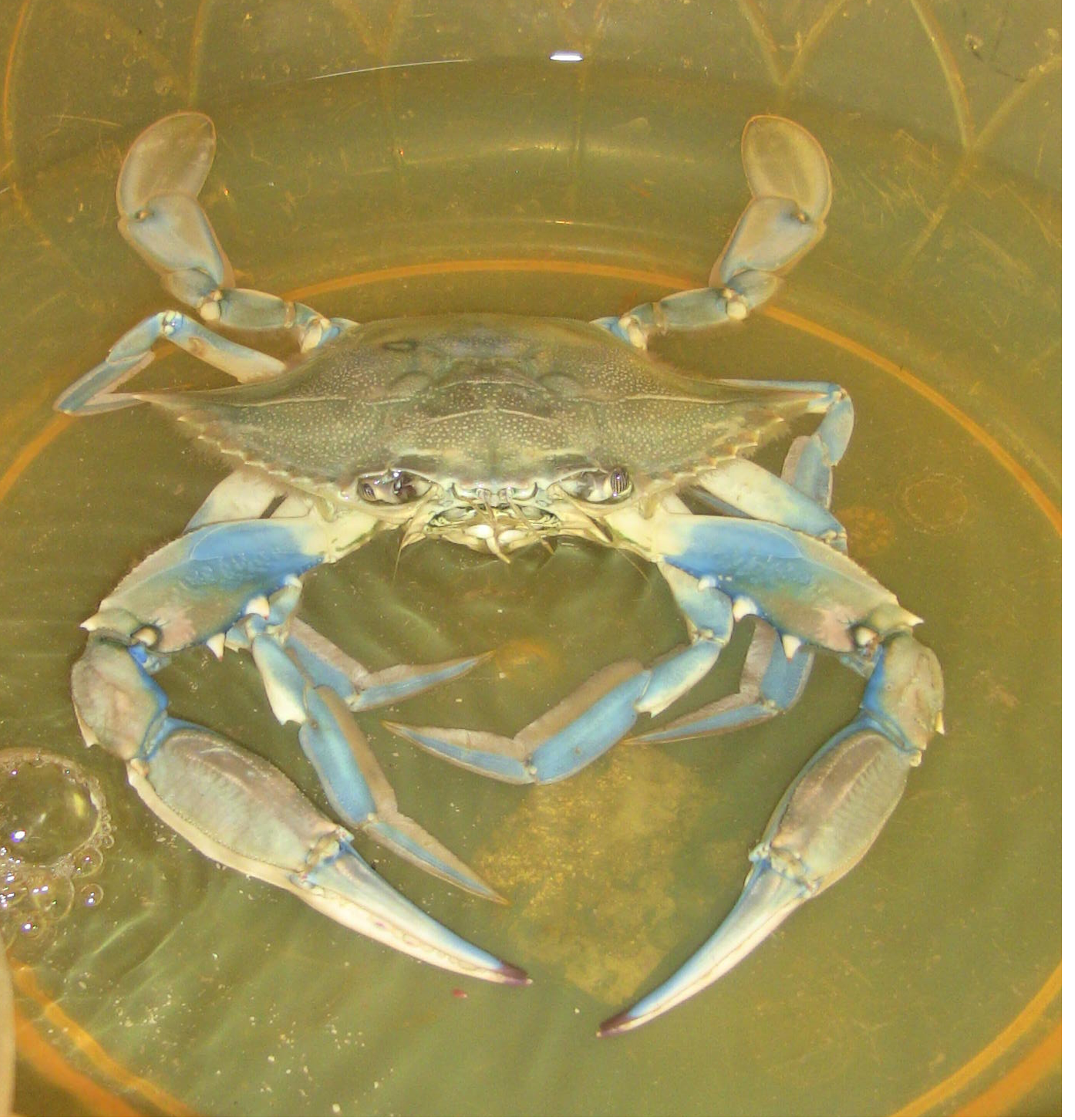

Supplement: S5 Fig — (TIF) [file pone.0148922.s005.tif]

## Slide 1
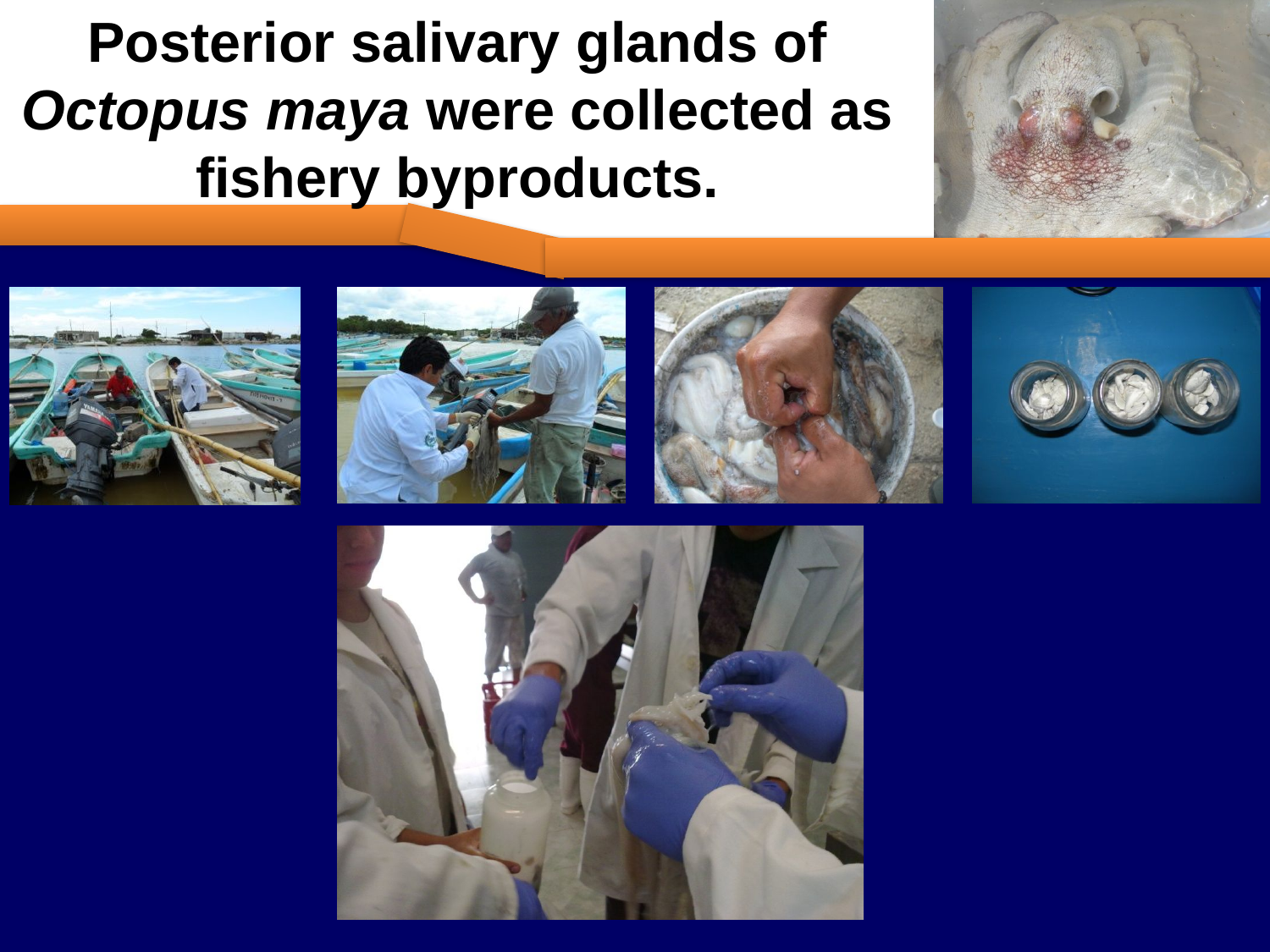

Posterior salivary glands of Octopus maya were collected as fishery byproducts.

Supplement: S1 Powerpoint — (PPTX) [file pone.0148922.s007.pptx]
